# Virulence Plasmid Modulates Glucose-Mediated Biofilm Regulation in *Yersinia enterocolitica*

**DOI:** 10.3390/life15111689

**Published:** 2025-10-30

**Authors:** Yunah Oh, Tae-Jong Kim

**Affiliations:** Department of Forest Products and Biotechnology, Kookmin University, Seoul 02707, Republic of Korea; oya010111@kookmin.ac.kr

**Keywords:** *Yersinia enterocolitica*, virulence plasmid, biofilm formation, glucose metabolism, extracellular polymeric substances, cyclic AMP

## Abstract

*Yersinia enterocolitica* is a foodborne pathogen capable of biofilm formation and virulence modulation in response to environmental signals. Among these, glucose—present at physiologically relevant concentrations in the human body—may serve as a regulatory cue affecting infection-associated pathways, including those governed by the pYV virulence plasmid. Although the role of glucose has been investigated under host-mimicking conditions, its impact in non-host environments remains poorly understood. This study was designed to evaluate the glucose-dependent physiological responses of two isogenic *Y. enterocolitica* strains, KT0001 (pYV-negative) and KT0003 (pYV-positive), under non-host conditions (26 °C). Both strains were cultured in TYE medium containing 0–3% glucose. Comparative analyses were conducted under identical in vitro conditions to elucidate plasmid-associated phenotypic differences. Glucose elicited markedly divergent responses. In KT0001, growth remained unaffected; however, biofilm formation declined by 77.7%, accompanied by a 90% reduction in surface hydrophobicity, a 40% decrease in motility, and a 59% drop in intracellular cyclic AMP—suggesting classical carbon catabolite repression. Conversely, KT0003 exhibited 86% growth inhibition but maintained biofilm levels. This was associated with substantial extracellular polymeric substance induction (~20-fold increase in polysaccharides and ~4.7-fold in extracellular DNA) and nearly fivefold elevation in cyclic AMP levels, despite concurrent decreases in motility (64%) and hydrophobicity (40%). These findings indicate that glucose functions as a strain-specific modulator in *Y. enterocolitica*. In particular, KT0003’s response suggests that the pYV plasmid enables the bacterium to interpret glucose as a host-associated cue, even under non-host conditions, potentially initiating virulence-related adaptations prior to host contact.

## 1. Introduction

Biofilms, composed of extracellular polymeric substances (EPS) such as polysaccharides, proteins, and eDNA, protect bacterial communities and facilitate environmental persistence [1]. This matrix provides structural integrity and establishes a physicochemical barrier that protects embedded cells from environmental stresses, including desiccation, oxidative agents, antimicrobials, and host immune responses [2,3,4]. Cells within biofilms exhibit altered gene expression and reduced metabolic activity compared to their planktonic cells, resulting in enhanced stress tolerance and long-term survival under adverse conditions [5]. Consequently, biofilm formation contributes not only to persistent infections in clinical treatments but also to the contamination of food-processing surfaces, where eradication remains a significant challenge [6].

*Yersinia enterocolitica* is a Gram-negative, psychrotrophic enteropathogen and the causative agent of yersiniosis, a zoonotic disease typically transmitted through contaminated food [7]. Its ability to replicate at low temperatures (0–4 °C) and to form robust biofilms on abiotic surfaces such as stainless steel and plastic makes it particularly problematic in cold-chain environments [8]. Within host tissues, *Y. enterocolitica* adheres to epithelial cells, where biofilm formation facilitates immune evasion and chronic colonization [9]. Although previous studies have elucidated environmental factors such as temperature and salinity as modulators of biofilm formation [10], the role of carbon sources—particularly glucose—remains poorly defined in this species.

Glucose functions not only as a major carbon and energy source but also as a metabolic signal that regulates a variety of bacterial behaviors, including virulence, motility, and community structure [11]. In model organisms like *Escherichia coli*, glucose availability is sensed through the phosphoenolpyruvate phosphotransferase system, leading to decreased cyclic AMP (cAMP) levels and subsequent repression of catabolite-sensitive genes, including those associated with biofilm development [12,13]. In contrast, some bacterial species, including *Pseudomonas aeruginosa* and *Staphylococcus aureus*, show enhanced biofilm formation in response to glucose, often through increased EPS synthesis or alterations in redox signaling and second messenger pathways [14,15].

Such divergent outcomes suggest the species- and strain-specific nature of glucose-mediated regulation. Indeed, emerging evidence suggests that even genetically similar strains may respond differently to glucose due to differences in accessory genetic elements and regulatory circuitry. In this context, the virulence plasmid pYV—a 70-kb element encoding the type III secretion system—is of particular interest. The pYV plasmid encodes multiple regulators such as VirF, YmoA, and YopN that may influence cell surface properties and glucose sensing. Beyond its well-established role in delivering *Yersinia* outer proteins into host cells [16], the pYV plasmid may exert broader regulatory influences on surface-related phenotypes, including motility, hydrophobicity, and biofilm development, particularly under nutrient-limiting conditions. However, its specific contribution to glucose-mediated physiological changes remains largely speculative. Previous studies in *Yersinia pestis* and *Y. pseudotuberculosis* also reported glucose-dependent modulation of biofilm formation, supporting the genus-wide relevance of this regulatory phenomenon [17,18].

Of particular note, glucose concentrations in human blood (~0.1–0.15%) may act not only as nutrients but also as environmental cues that signal host presence [19,20]. Despite extensive research on virulence regulation at 37 °C, the effects of glucose signaling at environmental temperatures remain poorly defined. For pathogens such as *Y. enterocolitica*, encountering such glucose-rich environments during early stages of infection may initiate virulence-related transitions. Despite this possibility, most previous studies have focused on glucose effects at 37 °C—mimicking host-like conditions—and little is known about how *Y. enterocolitica* interprets glucose signals in non-host environments, such as during early colonization on abiotic surfaces at lower temperatures. Distinguishing host and non-host conditions is critical because environmental adaptation to glucose may enhance bacterial persistence and transmission before infection.

In this study, we investigated how glucose modulates key physiological traits of *Y. enterocolitica* under non-host conditions (26 °C), focusing on the role of the pYV plasmid in mediating strain-specific responses. Using two isogenic strains—KT0001 (pYV-negative) and KT0003 (pYV-positive)—we examined glucose-induced changes in growth, biofilm formation, motility, cell surface hydrophobicity, EPS production, and intracellular cAMP levels. We hypothesized that the presence of the pYV plasmid reprograms glucose perception in *Y. enterocolitica*, promoting a biofilm-persistent phenotype even under non-host conditions. By characterizing these responses under identical in vitro conditions, we aimed to determine whether the presence of the pYV plasmid reprograms metabolic sensing and biofilm-associated behaviors in a glucose-dependent manner. Our findings provide new insights into how virulence-associated plasmids modulate environmental adaptability and may initiate pathogenesis even before host contact.

## 2. Materials and Methods

An overview of the experimental workflow is as follows: strain preparation, biofilm quantification, EPS analysis, hydrophobicity and motility assays, and biochemical quantification (HPLC and cAMP).

### 2.1. Bacterial Strains and Culture Conditions

Two isogenic strains of *Y. enterocolitica* were employed in this study: KT0001, a pYV-negative derivative of *Y. enterocolitica* KCCM 41657 (biotype 1B, serotype O:8), and KT0003, a pYV-positive strain constructed by introducing the virulence plasmid pYV100 via triparental conjugation [21]. The presence or absence of pYV was confirmed using plasmid-specific PCR and assessment of calcium-dependent growth. Both isogenic strains (KT0001 and KT0003) were cultured under identical conditions to ensure that observed differences reflect the influence of pYV plasmid presence. For routine maintenance, both strains were streaked on TYE medium made with 1% (*w*/*v*) Bacto Tryptone (catalog number: 211705; Thermo Fisher Scientific Korea Ltd., Seoul, Republic of Korea) and 0.5% (*w*/*v*) yeast extract (catalog number: 212750; Thermo Fisher Scientific Korea Ltd., Seoul, Republic of Korea) with 1.5% (*w*/*v*) Bacto agar (catalog number: 214010; Becton, Dickinson and Company, Sparks, MD, USA) to obtain a single colony. Pre-cultures were prepared by inoculating single colonies into TYE medium and incubating overnight at 26 °C with shaking at 200 rpm. For experimental assays, overnight cultures were diluted to an initial Absorbance at 600 nm (Abs_600_) of 0.05 in fresh TYE medium supplemented with D(+)-glucose (catalog number: 64220-0650; Junsei Chemical Co., Ltd., Tokyo, Japan) at final concentrations of 0%, 0.25%, 0.5%, 0.75%, 1%, 2%, or 3% (*w*/*v*). We used 0–3% glucose to reflect both physiological and nutrient-rich conditions, and incubations at 26 °C and 37 °C to represent environmental and host-like settings. Cultures were aliquoted into sterile 96-well polystyrene microplates and incubated under the indicated conditions for phenotypic analysis. Cultures were incubated for 24 h without antibiotic supplementation.

### 2.2. Biofilm Formation Assay

Biofilm formation was assessed using the crystal violet (CV) staining method with minor modifications from a previously described protocol [22]. Biofilms were grown in untreated polystyrene 96-well plates with 200 µL per well, covered during incubation to minimize evaporation. Bacterial cultures were incubated statically in TYE medium containing 0–3% (*w*/*v*) glucose at either 26 °C or 37 °C for 24 h. After incubation, planktonic cells were quantified by measuring Abs_600_, and non-adherent cells were removed by gentle washing with distilled water.

The remaining biofilms were stained with 1% (*w*/*v*) CV for 20 min at room temperature. After discarding excess stain, wells were rinsed thoroughly twice, and the bound CV was solubilized using 95% ethanol. Absorbance at 600 nm was recorded to quantify biofilm biomass. Uninoculated media were included as negative controls to correct for background staining. All experiments were performed with six independent biological replicates.

### 2.3. Quantification of Extracellular Polymeric Substances (EPS)

To evaluate the effect of glucose on EPS production, three major EPS components—proteins, polysaccharides, and eDNA—were quantified from cultures grown in TYE medium with or without glucose supplementation at 26 °C for 24 h, as previously described [21]. EPS was isolated by centrifugation (13,362× *g*, 10 min, 4 °C), followed by washing with phosphate-buffered saline (PBS) and precipitation with cold ethanol (−20 °C). EPS was extracted from biofilm biomass and normalized to cell density (Abs_600_). Ethanol precipitation efficiency was validated using a standard reference sample to ensure recovery consistency across assays. Protein content was determined using the Bradford assay (catalog number: BC1017-500-02; Biosesang Co., Ltd., Yongin, Republic of Korea), with bovine serum albumin (BSA) as the standard, and absorbance measured at 595 nm. Total polysaccharide content was assessed by the phenol–sulfuric acid method using D-glucose as a reference standard and measuring absorbance at 490 nm. Quantification of eDNA was performed using SYTOX™ Green Nucleic Acid Stain (Catalog number: S7020, Thermo Fisher Scientific Korea Inc., Seoul, Republic of Korea), with excitation and emission wavelengths set at 485 nm and 528 nm, respectively.

All EPS component values were normalized against cell density measured at Abs_600_ to correct for growth variability. Each measurement was conducted in three independent biological replicates.

### 2.4. Measurement of Residual Glucose

To determine the extent of glucose utilization by the bacterial strains, culture supernatants were collected after 24 h of incubation in TYE medium at 26 °C. Samples were clarified using Separa^®^ filter vials (catalog number: MV32ANPRC002GC01; GVS Korea Ltd., Namyangju, Republic of Korea) to remove cellular debris. Residual glucose concentrations were analyzed using a high-performance liquid chromatography (Arc HPLC system, Waters Korea Co., Gwangmyeong, Republic of Korea) with a refractive index detector (catalog number: 2414; Waters Korea Co.) using a Eurospher II 100-5 NH_2_ column (250 × 4.6 mm, 5 μm particle size, catalog number: 25EE190E2J; Knauer Wissenschaftliche Geräte GmbH, Berlin, Germany). Samples were filtered through 0.22 µm membranes prior to injection (20 µL) at 35 °C. The calibration range was 0.05–10 mM with a limit of detection (LOD) of 0.01 mM. The mobile phase consisted of acetonitrile/water (7:3, *v*/*v*) at a flow rate of 1.0 mL/min. Calibration curves were constructed using standard D(+)-glucose solutions. Uninoculated media controls were included to assess any non-biological glucose degradation. All measurements were carried out in triplicate.

### 2.5. Intracellular cAMP Quantification

To assess glucose-mediated changes in intracellular cAMP levels were measured using the cAMP-Glo™ Assay Kit (Catalog number: V1501; Promega Korea Ltd., Seoul, Republic of Korea) following the manufacturer’s protocol. Bacterial cultures grown in TYE medium containing various concentrations of glucose (0–3%, *w*/*v*) at 26 °C for 24 h were subjected to heat lysis by boiling for 5 min, followed by centrifugation (13,362× *g*, 10 min) to obtain cell-free extracts. Aliquots of the supernatant were used to prepare assay reactions. Luminescence was measured using a Synergy™ LX Multi-Mode Reader (BioTek Instruments Korea Ltd., Seoul, Republic of Korea). Quantification was achieved using a standard curve generated with known cAMP concentrations, and results were normalized to cell density (Abs_600_). Intracellular cAMP concentrations were further normalized to total protein content to account for biomass variability. Samples were treated with phosphodiesterase inhibitors prior to lysis to prevent cAMP degradation and analyzed in duplicate technical replicates for each biological replicate. Each assay was independently performed in triplicate.

### 2.6. Flagella Staining and Microscopy

Flagellar structures were visualized using a classical mordant-based staining method with slight modifications from the protocol of Kim et al. [10]. Briefly, bacterial cultures grown for 24 h at 26 °C in TYE medium, with or without glucose supplementation, were harvested and stained using a mordant solution comprising tannic acid, phenol, aluminum potassium sulfate, and crystal violet. Stained cells and flagella were examined under an AXIO Scope.A1 microscope (ZEISS Korea, Seoul, Republic of Korea) equipped with a 1000× oil immersion objective lens. For each condition, at least 100 individual cells were assessed to determine the proportion of flagellated cells. Microscopic observations were repeated in independent replicates to ensure reproducibility.

### 2.7. Motility Assay

Swimming motility was evaluated on soft TYE agar plates containing 0.3% Bacto agar. Desired glucose concentrations (0–3%, *w*/*v*) were added prior to medium solidification. Single bacterial colonies were inoculated into the center of the agar using sterile needles. After incubation at 26 °C for 24 h under static conditions, motility halos were imaged using the Gel Doc™ XR+ imaging system (Bio-Rad Laboratories Inc., Hercules, CA, USA), and halo diameters were quantified using Image Lab™ software ver 3.0 (Bio-Rad Laboratories Inc.). Motility halos were measured edge-to-edge to maintain consistency across all replicates. All measurements were conducted in triplicate and expressed as means ± standard deviations (SD).

### 2.8. Cell Surface Hydrophobicity Assay

The microbial adhesion to hydrocarbons (MATH) assay was employed to assess cell surface hydrophobicity, as previously described by Muthamil et al. [23], with minor adjustments. Following 24 h incubation, bacterial cells were collected, washed twice with PBS, and resuspended to an absorbance of Abs_600_ = 0.6 (denoted H_0_). n-Hexadecane was pre-equilibrated with PBS before use to minimize temperature-dependent variability. Samples were vortexed for 60 s at 26 °C to ensure consistent phase separation. A 10% (*v*/*v*) volume of n-hexadecane was added to the cell suspension, followed by vigorous vortexing for 1 min. After phase separation at room temperature for 15 min, the aqueous phase absorbance (*H*) was recorded. The hydrophobicity index (*HI*) was calculated using the formula: *HI* (%) = (*H*_0_ − *H*/*H*_0_) × 100. Each assay was performed in three independent replicates.

### 2.9. Statistical Analysis

All numerical data were processed using Microsoft Excel 2019 (Microsoft Korea Ltd., Seoul, Republic of Korea). Pairwise comparisons were analyzed using an unpaired, two-tailed Student’s *t*-test. For multiple group comparisons, one-way analysis of variance followed by Tukey’s post hoc test was applied. Normality was tested using the Shapiro–Wilk test, and variance homogeneity was verified using Levene’s test. Biological replicates refer to independent cultures prepared on different days. The results are presented as mean ± SD from a minimum of three independent experiments. Statistical significance was considered at *p* < 0.05, and highly significant differences were denoted at *p* < 0.01.

## 3. Results

### 3.1. Effects of Glucose on Cell Growth

**Figure 1:**
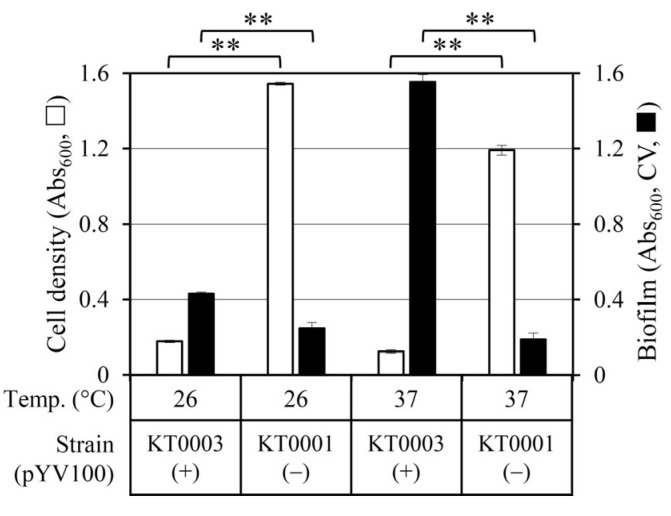
Comparative analysis of cell growth and biofilm formation in *Y. enterocolitica* KT0001 (pYV−) and KT0003 (pYV+) strains cultured for 24 h in TYE medium with 2% glucose at 26 °C and 37 °C. Cell density (white bars, Abs_600_) and biofilm biomass (black bars, Abs_600_ after crystal violet [CV] staining) are shown. Data represent mean ± standard deviation from three independent experiments. Statistical significance was determined using a Student’s *t*-test. Significant differences between data are denoted by ** for *p* < 0.01. A comparative summary of growth and biofilm data across both temperatures is provided in Supplementary Materials Table S1.

To assess the role of the pYV virulence plasmid and temperature on glucose-dependent physiology, two isogenic strains of *Y. enterocolitica*—KT0001 (pYV-negative) and KT0003 (pYV-positive)—were cultivated in TYE medium containing 2% glucose for 24 h at 26 °C and 37 °C. Bacterial growth and biofilm formation were quantified by measuring Abs_600_ and CV staining, respectively (Figure 1).

At 26 °C, KT0001 showed robust growth (Abs_600_ = 1.54), whereas KT0003 exhibited severe growth inhibition (Abs_600_ = 0.18). Despite lower cell density, KT0003 produced more biofilm than KT0001, suggesting that the presence of pYV plasmid promotes biofilm development under environmental conditions, possibly at the expense of active proliferation.

At 37 °C, the physiological gap between the strains became more pronounced. KT0001 maintained substantial growth (Abs_600_ = 1.19) and formed minimal biofilm (Abs_600_, CV = 0.19). In contrast, KT0003 showed near-complete growth suppression (Abs_600_ = 0.12) but significantly increased biofilm biomass (Abs_600_, CV = 1.55), indicating a temperature-induced phenotypic shift favoring biofilm formation.

These observations reveal a growth–biofilm trade-off in the pYV-positive strain, which is further amplified at host temperature. The findings suggest that pYV-encoded elements may mediate a glucose-responsive, temperature-dependent regulatory mechanism that prioritizes sessile lifestyle and survival over proliferation in host-like environments.

### 3.2. Glucose Supplementation Differentially Affects Planktonic Growth and Biofilm Formation Depending on Strain and Temperature

To further evaluate the influence of glucose under non-host and host-like conditions, the physiological responses of both strains were analyzed in TYE medium with 2% glucose and compared to previously reported data without glucose supplementation [21].

At 26 °C, glucose inhibited the planktonic growth of KT0003 (pYV-positive) by approximately 86% (Abs_600_: 1.28 → 0.18). Interestingly, despite this growth inhibition, biofilm formation increased by 72.4% (Abs_600_, CV: 0.25 → 0.431), suggesting a glucose-induced shift toward a biofilm-associated phenotype. In contrast, KT0001 (pYV-negative) showed a 6.2% increase in growth (Abs_600_: 1.45 → 1.54), accompanied by a marked 80.3% reduction in biofilm formation (Abs_600_, CV: 1.27 → 0.25), indicating a preferential change toward planktonic proliferation in the absence of virulence plasmid regulation.

At 37 °C, KT0003 maintained its biofilm-dominant phenotype, although both growth and biofilm production were slightly reduced in response to glucose (Abs_600_: 0.20 → 0.12; Abs_600_, CV: 1.90 → 1.55). For KT0001, glucose affected only minimal on either growth increasing modestly (Abs_600_: 0.95 → 1.19) and biofilm formation remaining nearly unchanged (Abs_600_, CV: 0.21 → 0.19).

These results indicate that glucose functions as a differential regulatory signal, inducing a shift toward a sessile lifestyle in KT0003 under environmental conditions, while favoring planktonic growth in KT0001 across temperatures. The divergent responses further emphasize the modulatory role of the pYV plasmid in balancing growth and adhesion-related phenotypes in response to metabolic cues.

### 3.3. Glucose Changes Growth and Biofilm Formation in a Concentration- and Strain-Dependent Manner at 26 °C

**Figure 2:**
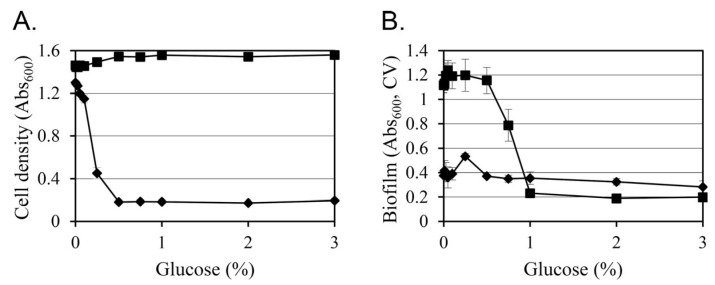
Glucose concentration-dependent changes in cell growth and biofilm formation of *Y. enterocolitica* strains KT0001 (pYV−, ■) and KT0003 (pYV+, ♦) cultured at 26 °C for 24 h. (**A**) Cell density assessed by absorbance at 600 nm (Abs_600_). (**B**) Biofilm biomass quantified by crystal violet staining (Abs_600_, CV). All data represent mean ± standard deviation from three biologically independent experiments.

Given that glucose supplementation had limited effects at 37 °C (Figure 1), subsequent analyses focused on 26 °C, where distinct glucose-responsive phenotypes were observed. To examine concentration-dependent changes, KT0001 (pYV-negative) and KT0003 (pYV-positive) were cultivated in TYE medium containing 0–3% glucose, and their planktonic growth and biofilm formation were monitored after 24 h (Figure 2).

KT0003 exhibited a clear dose-dependent growth inhibition. As glucose concentrations increased, Abs_600_ values decreased sharply from 1.30 (0%) to 0.17 (2%), indicating that glucose negatively affects the growth of pYV-positive cells even under nutrient-rich conditions. In contrast, KT0001 maintained consistent planktonic growth across all glucose levels, with Abs_600_ values ranging from 1.46 to 1.54, showing that the absence of the pYV plasmid confers glucose resistance in terms of growth suppression.

Biofilm production patterns also differed between strains. KT0003 showed relatively low and stable biofilm levels across glucose concentrations (Abs_600_, CV: 0.28–0.53), whereas KT0001 demonstrated a striking glucose-induced decline in biofilm formation, with Abs_600_, CV values dropping from 1.12 (0%) to 0.19 (2%). This represents an approximate 83% reduction, suggesting strong repression of surface-associated phenotypes by glucose in the plasmid-negative background.

These findings confirm that glucose exerts dual regulatory roles depending on strain background: it inhibits growth in the pYV-positive KT0003 while repressing biofilm development in the pYV-negative KT0001. The absence of such effects at host-like temperature further implies that glucose-mediated responses are finely tuned by both environmental conditions and plasmid-encoded regulatory elements.

### 3.4. Glucose Induces EPS Production in a pYV-Dependent Manner at 26 °C

**Figure 3:**
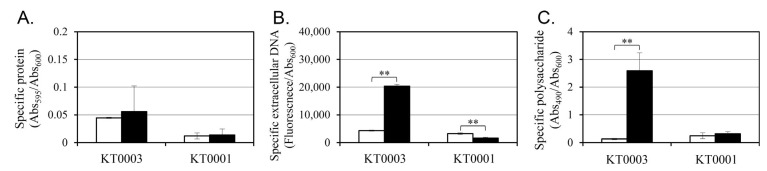
Quantitative analysis of EPS components in *Y. enterocolitica* strains KT0001 (pYV−) and KT0003 (pYV+) following 24 h incubation at 26 °C under glucose-free (white bars) and 2% glucose-supplemented (black bars) conditions. (**A**) Specific protein content, (**B**) Specific extracellular DNA (eDNA), (**C**) Specific polysaccharide content extracted from biofilm matrix. Data are presented as mean ± standard deviation from three independent biological replicates. Asterisks denote statistically significant differences (** *p* < 0.01).

To find whether glucose-induced changes in biofilm formation are associated with alterations in extracellular matrix composition, key components of the EPS—proteins, eDNA, and polysaccharides—were quantified in both KT0001 and KT0003 strains grown for 24 h at 26 °C, with or without 2% glucose (Figure 3). Data under glucose-free conditions are compared to our previous dataset [21].

In terms of protein content, KT0003 exhibited a slight increase following glucose supplementation (from 0.045 to 0.056), equivalent to a ~24% increment (Figure 3A). KT0001, however, remained unaffected by glucose, maintaining low protein levels (0.012 to 0.014). This suggests that while protein secretion is marginally stimulated by glucose in the pYV-positive strain, it is unlikely to be the primary factor driving enhanced biofilm formation. Although protein EPS differences were modest, they suggest altered matrix composition linked to plasmid presence.

The most prominent changes were observed in eDNA and polysaccharide levels. KT0003 showed a dramatic 4.7-fold increase in eDNA upon glucose addition (4334 to 20,332 AU), whereas KT0001 exhibited a 50% reduction (3247 to 1623 AU) (Figure 3B). This plasmid-dependent divergence highlights a potential role of eDNA in glucose-stimulated biofilm development specifically in the pYV-positive background.

Polysaccharide production was most strongly affected. In KT0003, glucose supplementation resulted in a ~20-fold increase (0.128 to 2.591), corresponding to an increase from 25 ± 3 µg/mL to 38 ± 4 µg/mL at 26 °C, while KT0001 showed only a modest rise (0.244 to 0.320) (Figure 3C). Given that polysaccharides are critical for structural integrity and cohesion within biofilms, this finding indicates that glucose significantly upregulates matrix-building capacity in KT0003, but not in KT0001.

These results demonstrate that glucose acts as a potent inducer of EPS biosynthesis in a pYV-dependent manner. In KT0003, both eDNA and polysaccharides respond robustly to glucose, supporting the formation of a stable biofilm matrix. In contrast, KT0001 lacks this response, reinforcing the conclusion that the pYV plasmid serves as a regulatory module enabling EPS upregulation under glucose-rich conditions. These data are consistent with earlier biofilm phenotypes (Figure 2) and provide further evidence that glucose may function as a signal for virulence-associated adaptation in pYV-positive strains.

### 3.5. Glucose Differentially Modulates Residual Sugar Levels and Intracellular cAMP in a Plasmid-Dependent Manner

**Figure 4:**
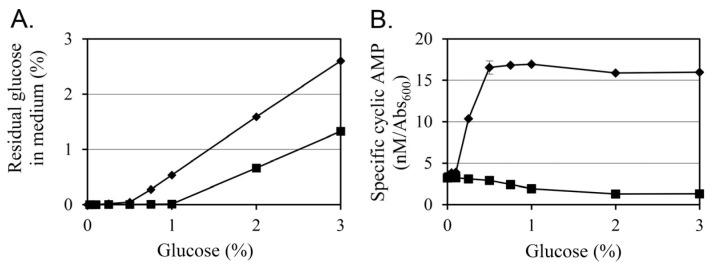
Effect of glucose concentration on residual glucose levels and intracellular cAMP concentrations in *Y. enterocolitica* strains KT0001 (pYV−) and KT0003 (pYV+). (**A**) Residual glucose in the culture supernatant after 24 h incubation at 26 °C, measured via high-performance liquid chromatography (HPLC). (**B**) Intracellular cAMP levels from the same cultures, normalized to Abs_600_ to adjust for differences in cell density. Strains were analyzed independently: KT0001 (■), KT0003 (♦). Data represent the mean ± standard deviation from three biological replicates.

To evaluate how glucose supplementation influences carbon metabolism and intracellular signaling in *Y. enterocolitica*, residual glucose concentrations in the culture supernatants and intracellular cAMP levels were measured after 24 h of growth at 26 °C under varying glucose concentrations (0–3%) (Figure 4).

The residual glucose analysis revealed a clear difference in metabolic utilization between the two strains (Figure 4A). In KT0003, residual glucose was undetectable at concentrations up to 0.5%, suggesting complete uptake under low-glucose conditions. However, when glucose was supplied at 0.75% or higher, accumulation of residual glucose was observed, indicating a threshold in metabolic processing capacity. In contrast, KT0001 showed a broader capacity for glucose utilization. Glucose was completely depleted up to 1% supplementation, and even at 2%, residual levels remained relatively low compared to KT0003. These results indicate that KT0001 metabolizes glucose more efficiently than KT0003, possibly due to its plasmid-independent energy metabolism and unimpeded catabolic regulation.

Intracellular cAMP levels further highlighted the physiological divergence between the two strains (Figure 4B). In KT0001, a typical pattern of catabolite repression was observed. The basal cAMP level under glucose-free conditions was 3.2 nM/Abs_600_, which decreased gradually with rising glucose concentrations and stabilized at approximately 1.3 under 2% glucose. This result aligns with the classical carbon catabolite repression (CCR) model, where elevated glucose reduces adenylate cyclase activity and consequently downregulates cAMP synthesis.

In contrast, KT0003 displayed an opposing trend. The cAMP level in glucose-free medium was comparable to KT0001 (3.4 nM/Abs_600_), but glucose addition resulted in a sharp increase in cAMP levels. At 0.5% glucose, cAMP rose markedly to 16.5 nM/Abs_600_ and remained elevated at higher concentrations. This result deviates from classical CCR and suggests that KT0003, harboring the pYV plasmid, activates an alternative regulatory mechanism that stimulates cAMP production in response to glucose availability. Such a response may support the induction of virulence-related or biofilm-associated pathways under glucose-rich environmental conditions.

These findings imply that KT0003 may employ a pYV-mediated reprogramming mechanism that uncouples cAMP regulation from conventional carbon sensing. This change in metabolic response could serve as a signal transduction mechanism that links extracellular glucose levels to adaptive phenotypes such as EPS production and sessile growth. The observed glucose-dependent increase in cAMP in KT0003 correlates well with the previously described EPS upregulation and biofilm enhancement, reinforcing the notion that the pYV plasmid facilitates a distinct physiological state favoring survival and colonization under nutrient-rich, non-host conditions.

### 3.6. Glucose Suppresses Flagellar Expression in a Strain-Independent Manner

**Figure 5:**
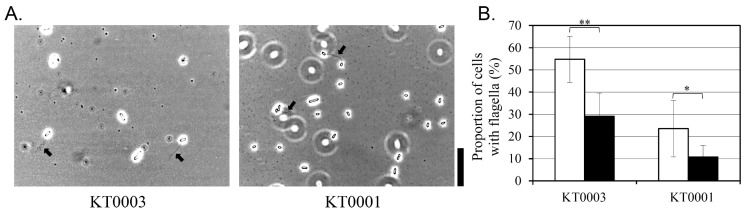
Impact of glucose on flagellar expression in *Y. enterocolitica* strains KT0001 (pYV−) and KT0003 (pYV+). (**A**) Representative microscopic images of flagella-stained cells grown under glucose-free conditions for 24 h at 26 °C. Arrows indicate cells with visibly stained flagella. (**B**) Proportion of flagellated cells quantified under glucose-free (white bars) and 2% glucose-supplemented (black bars) conditions. Values are presented as mean ± standard deviation from three independent biological replicates. Statistical significance was determined using a Student’s *t*-test. Significant differences between data are denoted by * for *p* < 0.05 and ** for *p* < 0.01. In panel (**A**), the black scale bar on the right indicates a length of 10 μm.

The influence of glucose on flagellar expression was examined by flagella staining in KT0001 (pYV-negative) and KT0003 (pYV-positive) strains grown for 24 h at 26 °C in the absence or presence of 2% glucose (Figure 5). The proportion of cells exhibiting visible flagella was quantified by microscopy.

In the absence of glucose, KT0003 showed a higher proportion of flagellated cells (55%) compared to KT0001 (24%), suggesting that the presence of the pYV plasmid correlates with increased flagellar expression under non-supplemented conditions. This observation may reflect an enhanced motility state associated with plasmid-encoded regulatory mechanisms, particularly under nutrient-limited environments.

Glucose supplementation significantly reduced the proportion of flagellated cells in both strains. KT0003 showed a 48% decrease, while KT0001 exhibited a comparable reduction of 54%. Importantly, structural integrity of the remaining flagella was maintained, indicating that glucose primarily affects the frequency or initiation of flagellar biosynthesis rather than the morphology of the flagellar apparatus itself. Reduced motility and decreased flagellation coincided with enhanced biofilm biomass, indicating a phenotypic switch toward a sessile lifestyle.

These results suggest that glucose exerts a suppressive effect on flagellar expression in *Y. enterocolitica*, regardless of pYV plasmid carriage. While the basal level of flagellation appears to be higher in the pYV-positive strain, the degree of glucose-mediated repression was similar across strains. This supports the notion that motility regulation in response to carbon availability is broadly conserved and may operate through classical catabolite repression pathways, independently or in parallel with virulence plasmid-associated signaling.

### 3.7. Glucose-Mediated Suppression of Motility

**Figure 6:**
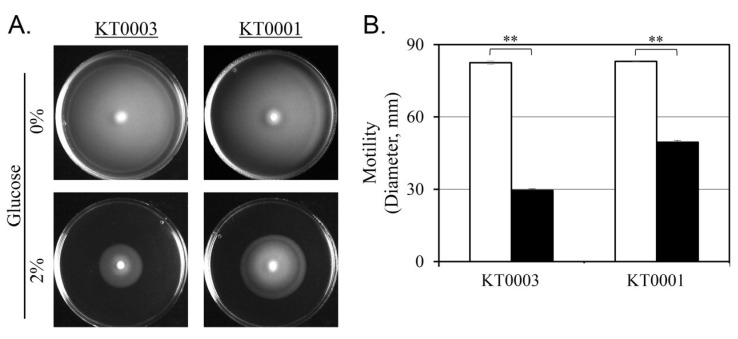
Glucose-dependent inhibition of swimming motility in *Y. enterocolitica* strains KT0001 (pYV−) and KT0003 (pYV+). (**A**) Representative images of motility halos formed on 0.3% soft agar plates after 24 h incubation at 26 °C under glucose-free and 2% glucose-supplemented conditions. (**B**) Quantitative analysis of motility zone diameters revealed significant suppression of motility by glucose in both strains, with KT0003 showing a more substantial reduction. Data are expressed as mean ± standard deviation from five independent biological replicates. White bars represent cultures grown without glucose (0%), whereas black bars indicate cultures supplemented with 2% glucose. ** *p* < 0.01.

To determine whether the observed repression of flagellar expression by glucose is reflected in functional motility, swimming assays were performed using soft agar medium containing 0.3% agar. KT0001 (pYV-negative) and KT0003 (pYV-positive) strains were inoculated and incubated at 26 °C for 24 h under glucose-free and 2% glucose-supplemented conditions (Figure 6).

In the absence of glucose, both strains exhibited active swimming behavior, forming wide and evenly distributed motility zones. The diameters of the motility zones were comparable between KT0001 (32.1 mm) and KT0003 (31.8 mm), indicating that basal motility capacity is similar regardless of plasmid carriage under glucose-free conditions.

Upon glucose supplementation, a marked decrease in motility was observed in both strains. KT0003 exhibited a more substantial reduction, with the motility zone shrinking to 29.5 mm—approximately 64% less than under glucose-free conditions. KT0001 also showed reduced motility, although to a lesser extent, with a 40% decrease to 49.5 mm (Figure 6B). These differences were statistically significant (*p* < 0.01), confirming that glucose represses swimming motility in both strains, albeit to different degrees.

The greater reduction in KT0003 aligns with earlier observations of decreased flagellar expression (Section 3.6) and enhanced cAMP accumulation (Section 3.5), suggesting that the presence of the pYV plasmid may amplify glucose sensitivity in motility-related pathways. These findings support the idea that glucose acts not only as a carbon source but also as a signal to modulate cellular behavior, particularly by promoting sessile states such as biofilm formation at the expense of motility—an effect more pronounced in virulent, plasmid-bearing strains.

### 3.8. Glucose-Induced Modulation of Cell Surface Hydrophobicity Differs by Strain

To examine whether glucose availability alters the surface physicochemical properties of *Y. enterocolitica*, we measured cell surface hydrophobicity using the MATH assay. This index serves as a functional indicator of surface properties that may influence adhesion potential and biofilm development.

**Figure 7:**
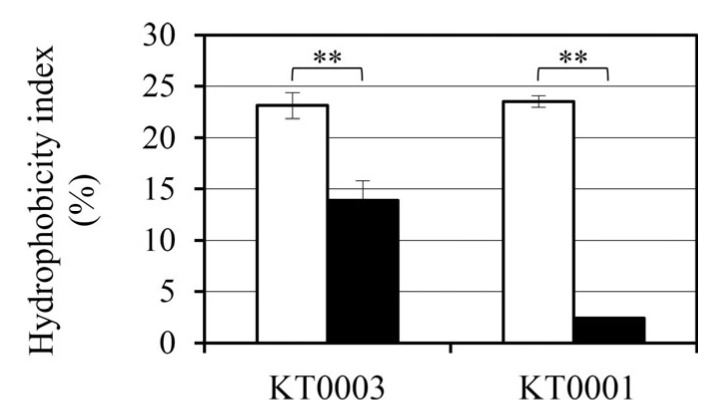
Glucose-induced reduction in cell surface hydrophobicity of *Y. enterocolitica*. Hydrophobicity of KT0001 (pYV−) and KT0003 (pYV+) was quantified using the MATH assay following 24 h incubation at 26 °C, in the presence or absence of 2% glucose. Both strains exhibited comparable hydrophobicity under glucose-free conditions. However, glucose supplementation significantly reduced hydrophobicity, with a more pronounced decrease observed in KT0001. Data represent the mean ± standard deviation of three independent experiments. White bars represent cultures grown without glucose (0%), whereas black bars indicate cultures supplemented with 2% glucose. ** *p* < 0.01.

In the absence of glucose, both KT0001 (pYV-negative) and KT0003 (pYV-positive) strains displayed comparable hydrophobicity indices, approximately 23%, suggesting that baseline cell surface characteristics are not strongly influenced by the presence of the pYV plasmid under nutrient-limited conditions (Figure 7).

Upon supplementation with 2% glucose, however, a marked reduction in hydrophobicity was observed in both strains, albeit with distinct magnitudes. KT0001 exhibited a dramatic decline of approximately 90%, reducing its hydrophobicity index to just 2.3%. KT0003 showed a more modest reduction of about 40%, with its hydrophobicity decreasing to 13.6%. Overall, the hydrophobicity index (*HI*) decreased from 0.42 ± 0.05 to 0.31 ± 0.04 under glucose supplementation. The term ‘hydrophobicity decrease’ is used consistently throughout the text for clarity. These values indicate that while both strains are responsive to glucose in terms of surface hydrophobicity, KT0001 is considerably more sensitive.

This differential response mirrors the trends observed in biofilm formation (Section 3.2), where KT0001 experienced a sharp decline under glucose-rich conditions, whereas KT0003 maintained a more stable biofilm profile. Given that surface hydrophobicity contributes to early adhesion and biofilm maturation, the pronounced reduction in KT0001 may partly explain its impaired biofilm development in glucose-supplemented environments.

These results suggest that glucose modulates surface properties in *Y. enterocolitica* in a strain-dependent manner. The presence of the pYV plasmid may confer a degree of resistance to glucose-mediated suppression of surface hydrophobicity, thereby supporting persistent adherence and biofilm formation under nutrient-rich conditions.

## 4. Discussion

This study provides a comparative physiological insight into how *Y. enterocolitica* responds to glucose availability under non-host conditions (26 °C), highlighting the physiological function of the pYV virulence plasmid in metabolic signaling, phenotypic reprogramming, and ecological adaptation. This study demonstrates that the pYV plasmid modulates glucose-mediated regulation of biofilm formation, EPS composition, and motility in *Y. enterocolitica*. By directly comparing two isogenic strains—KT0001 (pYV-negative) and KT0003 (pYV-positive)—we demonstrate that glucose triggers fundamentally divergent outcomes depending on plasmid carriage, revealing a broader regulatory role for pYV that extends beyond classical virulence gene expression.

In KT0001, the physiological response to glucose followed a classical CCR model: enhanced planktonic growth, decreased biofilm formation, reduced surface hydrophobicity, and downregulated motility. These effects were accompanied by a dose-dependent reduction in intracellular cAMP levels, consistent with established models in Enterobacteriaceae where glucose inhibits adenylate cyclase activity, thereby dampening cAMP-mediated transcription of catabolite-sensitive genes [24,25]. This classical repression cascade aligns with earlier studies in *Y. pseudotuberculosis* and *E. coli*, where high-glucose environments promote a motile, non-adherent state optimized for nutrient exploitation [26,27]. The inverse cAMP trend observed in KT0003 suggests that pYV may activate adenylate cyclase or inhibit phosphodiesterase activity, thereby maintaining elevated intracellular cAMP levels under glucose-rich conditions.

In contrast, KT0003 exhibited a non-typical, plasmid-dependent response to glucose. Glucose supplementation led to growth suppression, but paradoxically stimulated biofilm formation, elevated EPS production (notably eDNA and polysaccharides), and upregulated intracellular cAMP levels. The observed biofilm modulation arises primarily from glucose signaling, not nutritional enrichment, suggesting that glucose acts as a regulatory cue rather than a simple carbon source. This decoupling of glucose from CCR—markedly different from KT0001—suggests that pYV encodes or interacts with regulatory systems capable of overriding traditional carbon repression response. Unlike classical CCR in *E. coli*, glucose induced divergent cAMP trends in pYV^+^ cells, indicating uncoupled regulatory control that may involve plasmid-encoded factors or secondary messengers. Such inverted cAMP responses have not been previously reported in *Y. enterocolitica*, though studies in *Y. pestis* and other pathogens have suggested that virulence-associated regulators can modulate cAMP–Crp circuits under host-mimicking conditions [28]. Our findings extend this concept by identifying glucose as a potential host-associated signal that triggers a sessile, biofilm-dominant phenotype in pYV-positive strains even outside the host.

This hypothesis is further supported by the EPS profiling results. KT0003 exhibited striking upregulation of polysaccharide and eDNA secretion in glucose-rich conditions, while KT0001 remained largely unresponsive or showed suppression. As these EPS components are essential for structural biofilm stability, surface adherence, and immune evasion, the results imply that KT0003 prioritizes extracellular matrix development as a compensatory mechanism for reduced flagellar activity and surface hydrophobicity. Notably, previous work has shown that eDNA release is frequently associated with biofilm maturity and environmental resilience, particularly in pathogenic strains [21,29]. While *Y. pestis* exhibits similar EPS regulation, *Y. enterocolitica* shows plasmid-dependent variation, highlighting a genus-level divergence in biofilm regulatory strategies.

Motility repression was another key phenotypic divergence. Both KT0001 and KT0003 exhibited glucose-mediated reductions in flagellar expression and swimming zone diameters. However, only KT0003 retained biofilm formation capacity under these conditions, whereas KT0001 failed to maintain either motility or biofilm traits. This suggests that in KT0003, sessility is maintained through alternative mechanisms—likely EPS-driven—when motility is repressed, while KT0001 loses surface adherence altogether, reinforcing the role of pYV in lifestyle flexibility under carbon-rich stress. At 37 °C, these differences were attenuated, suggesting that plasmid-associated regulatory effects are more prominent under environmental conditions, whereas host-like temperatures may suppress or mask pYV-mediated modulation.

Differences in glucose metabolism efficiency further emphasize this regulatory contrast. KT0001 effectively depleted glucose from the medium at all tested concentrations, whereas KT0003 exhibited a saturation point beyond 0.5%, after which residual glucose remained unutilized. This may indicate that KT0003 limits catabolic activity than KT0001, possibly to prevent uncontrolled growth, conserve energy for matrix production, or avoid detection in host-like environments. Heroven & Dersch (2014) proposed similar adaptations in pathogenic *Yersinia*, where carbon flux is tightly regulated to balance virulence and survival [25].

Importantly, these observations were made at 26 °C—an environmental temperature where biofilm formation is ecologically relevant for foodborne transmission. Given that physiologically relevant glucose levels in the human body (~0.1–0.2%) are within the responsive range for KT0003, it is plausible that this strain detects glucose as a host-associated signal even outside the host. This preadaptive behavior may enhance environmental persistence on cold-chain surfaces, food processing equipment, or packaging materials, offering an evolutionary advantage over non-virulent strains. Enhanced persistence at 26 °C implies elevated risks in glucose-rich environments such as food residues, underscoring the importance of monitoring and controlling carbon source contamination in food-handling settings. Typical glucose concentrations in processed foods (1–3%) or clinical solutions (0.5–2%) fall within our tested range. Routine sanitation should therefore consider glucose-mediated persistence as a potential risk factor for contamination control.

All data presented in this study suggest that the pYV plasmid is more than a virulence determinant: it serves as a regulatory center that coordinates metabolic sensing, intracellular signaling, and phenotypic plasticity. While previous studies have primarily linked pYV to type III secretion and host cell invasion [16], our findings expand its functional scope to include modulation of CCR, cAMP regulation, EPS biosynthesis, and lifestyle transition toward a biofilm forming state. Such integration of nutritional signals with virulence readiness highlights a sophisticated regulatory network shaped by environmental selection pressures and horizontal gene transfer. Our findings support a broader view of pYV as a metabolic regulator that integrates virulence expression with environmental adaptation, positioning it as a key modulatory element in the ecological fitness of *Y. enterocolitica*.

KT0003 represents a stress-adaptive, biofilm-favoring variant of *Y. enterocolitica* that responds to glucose-rich conditions not with unchecked cell proliferation, but with metabolic restraint and matrix reinforcement—a strategy likely beneficial for survival in host-like or industrial environments. KT0001, lacking this regulatory flexibility, follows a growth-dominant trajectory incompatible with long-term surface attachment or virulence priming.

Importantly, the glucose-induced biofilm-promoting behavior observed in KT0003 has direct implications for food safety and clinical sanitation. Given that many food matrices and medical environments—such as enteral nutrition solutions, intravenous glucose infusions, or glucose-rich residues on processing surfaces—harbor physiologically relevant levels of glucose, pYV-positive strains like KT0003 may find these conditions particularly help to surface attachment and persistence. The enhanced EPS production and motility repression in KT0003 under such conditions would allow it to evade standard sanitation procedures, contributing to residual contamination and increased infection risk.

**Figure 8:**
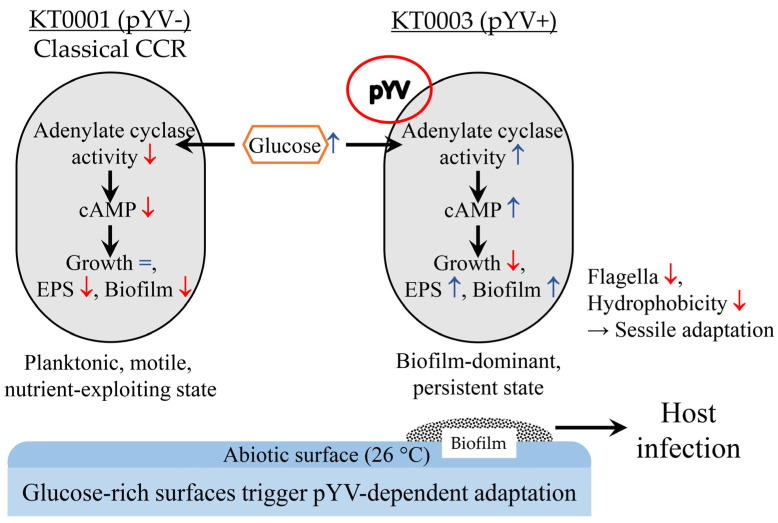
Conceptual model of pYV-mediated glucose signaling and biofilm regulation in *Yersinia enterocolitica.* In the pYV-negative strain (KT0001), glucose triggers classical carbon catabolite repression (CCR), reducing cAMP and biofilm formation while promoting planktonic growth. In contrast, the pYV-positive strain (KT0003) exhibits inverted glucose signaling, with elevated cAMP levels, enhanced EPS production, and reduced motility—driving a sessile, biofilm-persistent phenotype. The model illustrates how the pYV plasmid reprograms glucose perception to couple metabolic cues with virulence-associated adaptation, enhancing environmental persistence and infection potential. Red downward arrows (↓) indicate decreased levels or down-regulation, whereas blue upward arrows (↑) represent increased levels or up-regulation. The “=” symbol denotes no significant change in the corresponding parameter by glucose addition.

This points to a critical concern: glucose-rich environments may unintentionally select for virulent, plasmid-harboring strains by favoring biofilm-dominant lifestyles, while suppressing non-virulent counterparts like KT0001. In this view, glucose not only serves as a metabolic substrate but also functions as a selective pressure that enriches for pathogenic phenotypes capable of evading disinfection. Thus, in both food industry and clinical settings, especially those involving glucose exposure, sanitation protocols must account for the resilience of biofilm-forming, pYV-positive *Y. enterocolitica* strains. Failure to do so could facilitate their survival, dissemination, and ultimately, disease transmission—turning a simple nutrient into a biological function for pathogenic advantage. Although the molecular mechanism by which pYV uncouples cAMP from CCR remains unknown, further transcriptomic and mutagenic analyses are warranted to elucidate the underlying regulatory interactions. We propose a conceptual model linking glucose sensing, pYV plasmid signaling, EPS production, and enhanced biofilm survival (Figure 8), which collectively illustrate how metabolic cues are integrated into virulence-associated adaptive responses in *Y. enterocolitica*. This integrative perspective highlights the interplay between metabolism, virulence, and environmental adaptation in shaping bacterial survival, emphasizing that regulatory convergence across these domains underlies the ecological success of plasmid-bearing strains.

## 5. Conclusions

This study reveals that glucose modulates the physiology of *Y. enterocolitica* in a plasmid-dependent manner. The pYV-negative strain KT0001 exhibited a classical carbon catabolite repression pattern—enhanced growth, suppressed biofilm formation, and reduced cAMP levels. In contrast, the pYV-positive KT0003 responded to glucose with inhibited growth but increased biofilm formation, elevated EPS production, and enhanced cAMP signaling. These findings suggest that the pYV plasmid reprograms glucose perception, enabling a host-adaptive shift toward persistence rather than proliferation. Such metabolic–virulence integration highlights the plasmid’s broader role beyond toxin expression and may inform targeted strategies for biofilm control in pathogenic *Yersinia*. This study underscores that glucose-rich environments may favor plasmid-positive, biofilm-forming *Y. enterocolitica* populations, impacting both food safety and infection risk. Future research should elucidate the molecular basis of pYV-mediated glucose signaling and evaluate its ecological implications.

## Data Availability

The original contributions presented in this study are included in this article/Supplementary Materials. Further inquiries can be directed to the corresponding author.

## Supplementary Materials

The following supporting information can be downloaded at: https://www.mdpi.com/article/10.3390/life15111689/s1, Table S1: Comparative summary of planktonic growth and biofilm formation of *Yersinia enterocolitica* KT0001 (pYV−) and KT0003 (pYV+) in TYE medium ± 2% glucose after 24 h incubation at 26 °C and 37 °C.

## Abbreviations

The following abbreviations are used in this manuscript:

Abs_600_	Absorbance at 600 nm
cAMP	Cyclic AMP
CCR	Carbon catabolite repression
CV	Crystal violet
eDNA	Extracellular DNA
EPS	Extracellular polymeric substance
HI	Hydrophobicity index
HPLC	High-performance liquid chromatography
MATH	Microbial adhesion to hydrocarbons
PBS	Phosphate-buffered saline
SD	Standard deviation
