# Supplementary material for: Virulence Plasmid Modulates Glucose-Mediated Biofilm Regulation in Yersinia enterocolitica
Source: Life (Basel). 2025 Oct 30;15(11):1689. doi: 10.3390/life15111689 (PMC12653292; doi:10.3390/life15111689)
Supplement: Supplementary file 1 [file life-15-01689-s001.zip › life-3950667-supplementary.pdf]

Supplementary Materials Table S1. Comparative summary of planktonic growth and biofilm formation of *Yersinia enterocolitica* KT0001 (pYV–) and KT0003 (pYV+) in TYE medium ± 2 % glucose after 24 h incubation at 26 °C and 37 °C.

Strain	Temperature (°C)	Glucose (%)	Planktonic growth (Abs_{600} ± SD)	Biofilm biomass (Abs_{600} ± SD, CV)	Fold change in growth	Fold change in biofilm	Statistical significance[1]
KT0001 (pYV–)	26	0	1.45 ± 0.08	1.27 ± 0.09	—	—	—
		2	1.54 ± 0.07	0.25 ± 0.03	+1.06	0.20	$p < 0.01$
	37	0	0.95 ± 0.06	0.21 ± 0.02	—	—	—
		2	1.19 ± 0.04	0.19 ± 0.01	+1.25	0.90	n.s.
KT0003 (pYV+)	26	0	1.28 ± 0.05	0.25 ± 0.02	—	—	—
		2	0.18 ± 0.02	0.43 ± 0.04	0.14	+1.72	$p < 0.01$
	37	0	0.20 ± 0.03	1.90 ± 0.12	—	—	—
		2	0.12 ± 0.02	1.55 ± 0.10	0.60	0.82	$p < 0.05$

[1] Statistical significance for the change of biofilm formation by 2% glucose determined by unpaired, two-tailed *t*-test comparing ± 2 % glucose within each strain/temperature pair. n.s., not significant.
